# Characteristics of the obesogenic environment around schools are associated with body fat and low-grade inflammation in Brazilian children

**DOI:** 10.1017/S1368980023001696

**Published:** 2023-11

**Authors:** Mariana De Santis Filgueiras, Milene Cristine Pessoa, Josefina Bressan, Aline Siqueira Fogal Vegi, Ariene Silva do Carmo, Fernanda Martins de Albuquerque, Danielle Soares Gardone, Juliana Farias de Novaes

**Affiliations:** 1 Department of Nutrition and Health, Universidade Federal de Viçosa, Av. P.H. Rolfs s/n, Centro de Ciências Biológicas II, Campus Universitário, Viçosa 36570-900, Minas Gerais, Brazil; 2 Department of Nutrition, Nursing School, Universidade Federal de Minas Gerais, Av. Prof. Alfredo Balena, 190, Santa Efigênia, Belo Horizonte 30130-100, Minas Gerais, Brazil; 3 Nutrition School, Universidade Federal de Ouro Preto, Rua Dois, Campus Morro do Cruzeiro, Ouro Preto 35400-000, Minas Gerais, Brazil

**Keywords:** Adiposity, Adipokines, Inflammation, Built environment, Food environment

## Abstract

**Objective:**

To assess the association of obesogenic environmental characteristics around schools with body adiposity and adipokine concentrations in Brazilian children.

**Design:**

Cross-sectional study. Body adiposity was assessed using the dual-energy X-ray absorptiometry. Concentrations of leptin, adiponectin, retinol-binding protein 4 (RBP4) and chemerin were measured. Predominantly ultra-processed food (UPF) stores, public physical activity (PA) facilities, green spaces, walkability, traffic accidents and crime were evaluated. The neighbourhood unit was the 400 m (0·25 miles) road network buffer around schools. The association of environmental characteristics with body adiposity and adipokine concentrations was assessed by linear regression models using generalised estimating equations.

**Setting:**

Urban schools (*n* 24), Viçosa, Minas Gerais, Brazil.

**Participants:**

Children aged 8 and 9 years (*n* 378).

**Results:**

A higher density of predominantly UPF stores and a lower percentage of green space were associated with higher total (*β*: 0·12; 95 % CI 0·06, 0·18 and *β*: –0·10; 95 % CI –0·16, –0·04, respectively) and android body fat (*β*: 0·28; 95 % CI 0·13, 0·43 and *β*: –0·18; 95 % CI –0·32, –0·04, respectively). In addition, the densities of PA facilities and crime were inversely associated with leptin concentrations. Traffic accidents density and percentage of green spaces around schools had, respectively, a positive and an inverse association with concentrations of adiponectin and RBP4.

**Conclusions:**

Obesogenic environmental characteristics around schools were associated with total and android body fat, as well as with pro-inflammatory adipokine concentrations in Brazilian children from a medium-sized city.

Obesity is characterised by accumulation of adipose tissue, which is associated with adipokine concentrations, such as the higher leptin, retinol-binding protein 4 (RBP4) and chemerin (pro-inflammatory adipokines), and lower adiponectin (anti-inflammatory adipokine)^(1)^. It is known that leptin and adiponectin are positively and inversely associated, respectively, with diastolic blood pressure and homoeostasis assessment model of insulin resistance^(2)^. In addition, RBP4 and chemerin have been positively associated with total cholesterol and fasting blood glucose, respectively, demonstrating that adipokines may be important in the phenotyping of childhood obesity^(2)^.

Obesity and low-grade inflammation are multifactorial conditions resulting from the interaction of biological, social and environmental factors^(3)^. Obesogenic environment, defined as ‘the sum of influences that the surroundings, opportunities, or conditions of live have on promoting obesity in individuals or populations’^(4)^, may facilitate unhealthy food choices and/or sedentary behaviours^(5)^, which are related to obesity and inflammation pathways. Therefore, environmental factors have been identified that could contribute to obesity and related inflammation, including built food environments, physical activity (PA)-related environments and safety and crime-related factors^(6–8)^.

The availability and/or density of predominantly ultra-processed food (UPF) stores around schools, which are stores where the purchase of UPF represents more than 50 % of all purchases^(9)^, could influence the prevalence of obesity in children and adolescents^(6)^. However, in a review that included thirty-one studies, four identified an inverse association of proximity or density of food stores around schools (mainly fast food restaurants, convenience stores and grocery stores) with overweight and obesity in the paediatric population, while thirteen showed a lack of association^(10)^. The authors highlighted factors that make it difficult to reach a conclusion about the real influence of the presence of food stores near to schools and the nutritional status of children and adolescents, such as differences in the methods of classification, location and analysis of food stores^(10)^.

The poor infrastructure and insecurity from traffic and crime have been associated with less walking as a transport mode and the availability and/or access to PA facilities^(11,12)^. Furthermore, some studies have shown an inverse association of green spaces^(13)^, walkability^(14)^ and PA facilities^(15)^ with obesity in children and adolescents.

Most of these studies evaluated environmental factors around schools with childhood obesity assessed by BMI^(6,14)^. Although BMI is an easily obtained and low-cost measure, obesity phenotyping can be performed using more accurate methods, such as dual-energy X-ray absorptiometry (DXA), and by biomarkers, such as adipokines^(16)^.

The relationship of the obesogenic environment with adiposity and inflammatory markers is still poorly investigated among young people, in particular around schools. The interaction of children with schoolmates at school entrance and exit times could contribute for food choices and for physical and leisure activities^(17)^. Beyond to their households, children spend most of their time in schools, which are favourable places for the implementation of public policies aimed at promoting healthy food choices and PA, such as the regulation of food stores and the implementation of public facilities that promote physical and leisure activities around schools.

Additionally, few studies have evaluated characteristics of the obesogenic environment around schools in children from middle-income countries^(18,19)^. The rapid urbanisation that has occurred in some countries, such as Brazil, may have intensely modified eating habits and lifestyle^(20)^. Therefore, it is important to understand the role of the environment in markers of body adiposity and inflammation to promote actions for the prevention and control of childhood obesity and its comorbidities.

Given this, we aimed to evaluate the association of the obesogenic environmental characteristics around schools with body adiposity and adipokine concentrations in children from medium-sized Brazilian city. We hypothesised that an environment with obesogenic characteristics would be associated with the higher body adiposity measures and pro-inflammatory adipokine concentrations.

## Methods

### Study design, setting and sample

**Fig. 1:**
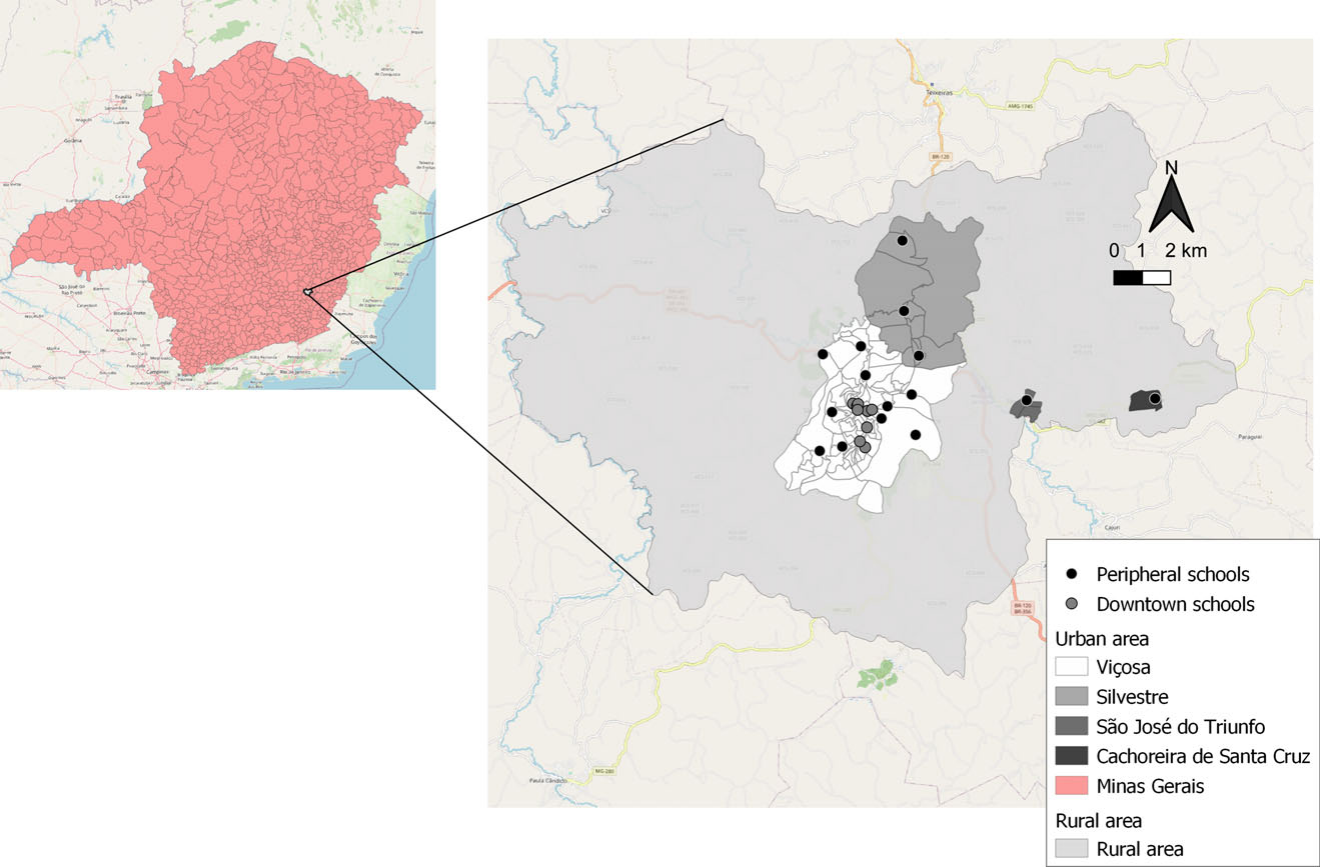
Urban area and districts of Viçosa, Minas Gerais, Brazil, 2015

This is a cross-sectional study conducted in 2015 with children aged 8 and 9 years enrolled in all schools in the urban area of Viçosa, Minas Gerais, Brazil, in its four districts: Viçosa, Silvestre, São José do Triunfo and Cachoeira de Santa Cruz (Fig. 1). According to the 2010 Census, the city has an area of 299 km^2^, 72 244 inhabitants, of which 93·2 % live in urban areas, and the gross domestic product per capita is US$ 4682·22 (R$ 19 869·94)^(21)^. Viçosa is a medium-sized city located in a mountainous valley with predominantly rugged terrain. It is characterised by being a university city and having a more urban than the agricultural economy, highlighting the role of the tertiary sector (commerce and services) as the main economic activity.

This study is a part of two major surveys. First, children’s data were obtained from the Schoolchildren Health Assessment Survey (*Pesquisa de Avaliação da Saúde do Escolar*, PASE, in Portuguese), an investigation carried out in 2015 that aimed to assess the cardiovascular health of children aged 8 and 9 years in Viçosa. This city had seventeen public and seven private schools in the urban area, with 1464 enrolled children between 8 and 9 years. From this population, the sample size was calculated, considering a confidence level of 95 %, an expected prevalence of 50 % (since the study considered multiple body fat measures and adipokines as outcomes) and an addition of 10 % to compensate for collection losses and 10 % to cover confounding factors, resulting in a minimum sample of 366 children. The number of children to be sampled in each school was proportional to the total number of students in each one. The selection of students in the school was conducted at random, until the total number of students needed from the twenty-four urban schools was completed. Children with the following characteristics would not be included: those with health problems that altered their nutritional status or body composition; chronic use of medicine that affected the glucose and/or lipid metabolism and when the parents/guardians could not be contacted after three attempts. At the end of the data collection, there were no losses of participants due to the non-accomplishment of all stages of the investigation. All 378 children from the survey were included in the analyses.

The second survey, ‘Data collection of the built environment of the urban area of Viçosa (MG)’, was conducted from December 2015 to July 2016 and provided information on built environment. The researchers carried out the data collection as an audit at food stores and public PA facilities.

These surveys were conducted according to the guidelines established in the Declaration of Helsinki and approved by the Human Research Ethics Committee of the *Universidade Federal de Viçosa* (UFV) (reference numbers 663.171/2014 and 1.821.618/2016). They were also approved by the Municipal Secretary of Education, the Regional Superintendent of Education and school boards. In addition, all parents/guardians signed an informed consent form. Participants were provided with a nutritional assessment and guidelines for health and nutrition after the study was completed. Participating schools also received a summary of the health status of the students participating in the research.

### Body adiposity markers

Body adiposity measures were assessed by a specialised technician using the DXA method (Lunar Prodigy Advance, GE Medical Systems Lunar) in the diagnostic imaging sector of the UFV Health Center. Due to the low radiation dose, DXA is a safe reference method for assessing body composition in childhood. The children were wearing light clothing, without metal and lying-in supine position on a stretcher, until the equipment completed the reading. Then, a report was generated with information on total and android body fat (%). The android region is between the ribs and the pelvis, totally enclosed by the trunk region.

### Inflammatory markers

After a 12-h fast, the blood samples were collected in the clinical analysis sector of the UFV Health Center by venipuncture in the antecubital region. The serum and plasma were separated and stored in 1·5 ml Eppendorf tubes at –80°C. We analysed concentrations of leptin, RBP4, adiponectin and chemerin.

Leptin serum was determined by an enzyme immunoassay method, with CV intra-assay < 13·3 % and inter-assay < 12·7 % (KAP2281, DIAsource®; standardised protocols from *Diagnósticos do Brasil*). Plasma concentrations of RBP4, adiponectin and chemerin were quantified by commercials ELISA sandwich kits, with coefficients of variation intra-assay < 10 % and inter-assay < 12 % (human RBP4: SEA929Hu; human adiponectin: SEA605Hu; human chemerin: SEA945Hu, Cloud Clone Corp.®; standardised protocols from *Laboratório Especializado em Análises Científicas* (LEAC)).

### Obesogenic environmental characteristics

Road network buffer was the neighbourhood unit used to assess the obesogenic environment around schools. Using the addresses of each school, we obtained buffers of 400 m (0·25 miles). It is suggested to use a 400 m buffer corresponding to a 5 min’ walk^(22)^. The 400 m buffer was chosen because children could walk a shorter distance around the school and Viçosa has a rugged terrain^(23)^.

We performed an objective assessment of the urban food environment and public PA facilities in the city. Using urban census tracts maps, trained researchers walked every street. A specific questionnaire was filled out when identifying a food store and a public PA facilities (squares, places for walking, among others).

For food stores, an audit was carried out inside the stores, using four types of questionnaires, adapted from a tool developed in Brazil, as follows: (a) objective assessment tool for food stores for household consumption; (b) objective assessment tool for food stores for immediate consumption; (c) objective assessment tool for mobile food vendors and (d) objective assessment tool for farmer’s markets^(24)^. The researchers identified the type of store (online Supplementary Material 1) and which products were sold (such as UPF – stuffed biscuits, snacks, sweets, frozen and sausage foods, sugar-sweetened beverages; sandwiches; bakery products; dairy products; fruits and vegetables). These food stores were grouped according to the list of Technical Study ‘Mapping Food Deserts in Brazil’ by Interministerial Chamber of Food and Nutritional Security (*Câmara Interministerial de Segurança Alimentar e Nutricional* (CAISAN), in Portuguese), which considers the degree of food processing according to the NOVA classification system (online Supplementary Material 1) for each Brazilian state and region^(9)^. Stores whose UPF represents more than 50 % of the total products available for sale were grouped as predominantly UPF stores^(9)^. Mobile unhealthy food vendors were including in the group because the audit allowed identifying the high sale of sweets and snacks.

Public PA facilities were assessed using the adapted Physical Activity Resource Assessment (PARA) instrument^(25)^. Any of the following structures present in the streets or public spaces were included: park, square, outdoor gym, public sports field or court, playground, walking/running track and bicycle track. The geographical coordinates (latitude and longitude) were obtained for each food store and public space.

The green spaces were obtained from the georeferenced database at the Brazilian Institute of Geography and Statistics (*Instituto Brasileiro de Geografia e Estatística* (IBGE), in Portuguese)^(26)^. This database contains information if, on the face of the block at observation or its front face or the median strip, there was a green space, that is, if there was a tree along the sidewalk and/or on a median strip that separates opposing lanes of traffic on divided roadways, even if only in part. The presence of green space was also considered when existing in areas without paving and/or without sidewalk. The percentage of presence in the census tract was determined by dividing the total number of households that had the characteristic by the total number of households in the tract and multiplying by 100.

To obtain the walkability index, data from the urban area of Viçosa were used to select a parsimonious variable set through factor analysis, with the principal components’ extraction method and varimax rotation with Kaiser normalisation. Thus, the distribution of each environmental variable that remained in the first factor (residential density, presence of sidewalk (%), commercial density, presence of public lighting (%) and the density of intersections between streets (connectivity)) was transformed into a z-score and summed to calculate the walkability index^(27)^.

Traffic accident was any harmful event that involves the vehicle, the road, the man and/or animals and to characterise it, it is necessary the presence of two of these factors^(28)^. Traffic accidents with victim were those that cause bodily injury or death. Considering the crimes, information was obtained on type of crimes: extortion, rape, theft, robbery, murder, bodily injury, kidnapping and false imprisonment. Each occurrence in 2015 had its geographical coordinates (latitude and longitude) informed by the Secretary of Public Security of the State of Minas Gerais. For the analyses, all traffic accidents (with and without victims) and types of crime were grouped.

Each school’s geographic coordinates (latitude and longitude) were collected from addresses using the Google Maps online search service (https://www.google.com/maps?hl=en). All characteristics presented in geographical coordinates were reprojected from the WGS 84 Geographic Coordinate System to the Projected Coordinate System, Universal Transverse Mercator System (UTM), 23S spindle, SIRGAS 2000 datum, using ArcGIS Pro (ESRI).

Predominantly UPF stores, PA facilities, traffic accidents and crime were counted within the 400 m buffer around the schools. For analysis purposes, we calculated the density of the values per 1000 inhabitants inside the buffer. The averages of inhabitants, green spaces (%) and walkability (z-score) in the census tracts inserted in each buffer around schools were calculated. All environmental variables were categorised according to the 50th percentiles of the sample.

### Adjustment variables

Individual and family information, such as age, sex, per capita income (US$) and screen time (> 2 h/d)^(29)^, were collected through a semi-structured questionnaire. Per capita income was calculated by dividing the total household income by the number of dependent residents on that income at household.

The mother’s BMI was calculated from weight and height, which were measured by an electronic digital scale with a capacity of 150 kg and a sensitivity of 50 g (Tanita®, model BC 553) and a vertical stadiometer, divided into centimetres and subdivided into millimetres (Alturexata®), respectively.

The school location (downtown/periphery) was evaluated, and the neighbourhood income was obtained from the average monthly per capita income of the census tracts inserted into the buffer around the school. Based on information from the 2010 Census^(26)^, the income and inhabitants of the census tracts were obtained to calculate the per capita income. Once the census tracts included in the buffer around the school were identified, the average per capita income of the neighbourhood was calculated.

### Statistical analysis

The distribution of variables was assessed using the Shapiro–Wilk test. Descriptive analysis of individual and environmental characteristics was performed by calculating frequency distributions for categorical variables and mean and sd for continuous variables.

For the spatial analyses, choropleth maps were created to visualise the spatial distribution of the schools according to localisation (downtown or periphery) and the densities of environmental characteristics around schools according to the 50th percentile.

To assess the association between variables of obesogenic environment (independent variables) and body adiposity and adipokine concentrations (dependent variables), linear regression models were performed with generalised estimating equations, which considers the aggregate effect of individuals (schools). We used the exchangeable correlation structure, which is recommended when the observations are grouped into some specific structure, assuming that the correlation of observations between individuals in a group is the same. The variables were treated as gamma distribution, with a log link function. Models were adjusted by age, sex, screen time, per capita income, mother’s BMI, school location (downtown/periphery) and neighbourhood income.

Unstandardised coefficients and their respective confidence intervals (95 % CI) were calculated. Data analyses were performed using the statistical software Stata® version 14 (StataCorp LP). The level of significance for all statistical tests was 5 %.

## Results

**Fig. 2:**
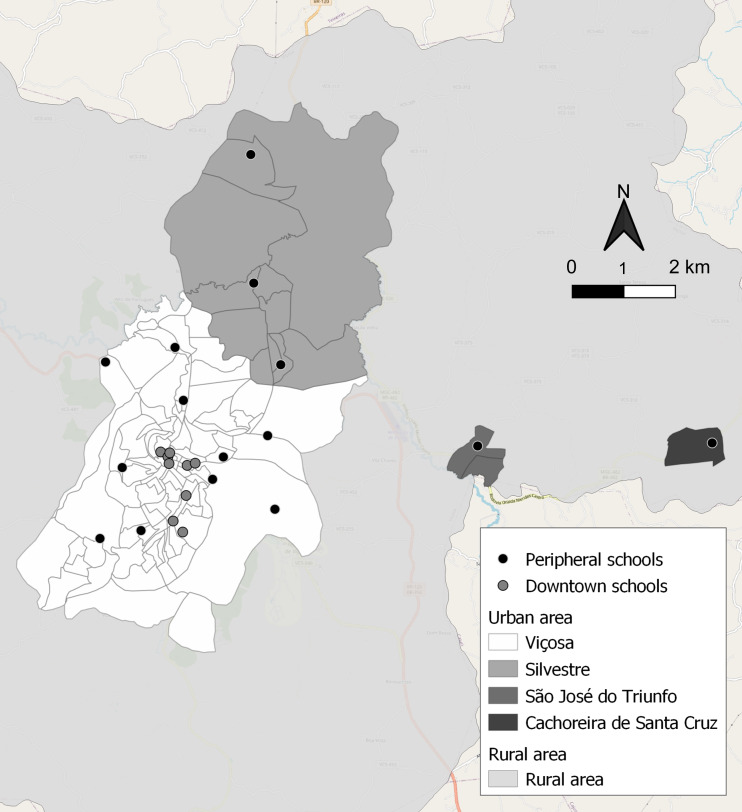
Geospatial distribution of urban schools in downtown and peripheral areas. Viçosa, Minas Gerais, Brazil, 2015

Considering the individual characteristics, participants’ mean ± sd age was 8·5 ± 0·5 years, and 52·1 % were girls. The means ± sd of total and android body fat (%) were, respectively, 24·2 ± 10·1 and 17·7 ± 12·2. The means ± sd of leptin, RBP4, adiponectin and chemerin concentrations were, respectively, 5·7 ± 9·5 ng/ml, 4·0 ± 0·7 μg/ml, 13·6 ± 7·4 μg/ml and 83·6 ± 64·8 ng/ml. Among the twenty-four evaluated schools, 20 (83·3 %) were in the Viçosa district, two (8·3 %) in Silvestre, one (4·2 %) in São José do Triunfo and one (4·2 %) in Cachoeira de Santa Cruz, 70·8 % (*n* 17) were public and 37·5 % (*n* 9) were in downtown (Fig. 2).

**Fig. 3:**
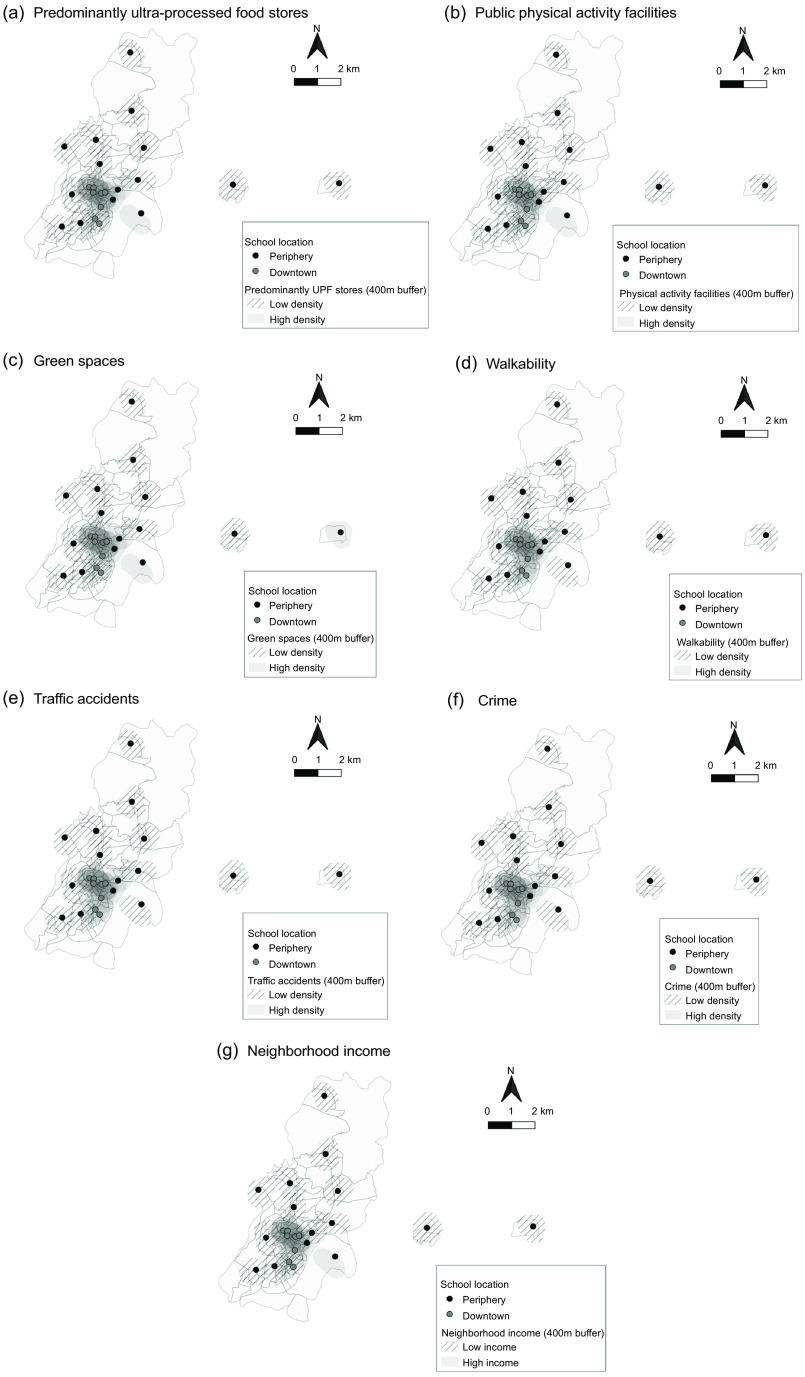
Densities of predominantly ultra-processed food (UPF) stores (a), public physical activity facilities (b), green spaces (c), walkability (d), traffic accidents (e), crime (f) and neighbourhood income (g) around urban schools. Viçosa, Minas Gerais, Brasil, 2015

We identified 612 food stores in whole city, of which 43·6 % (*n* 267) were predominantly UPF (52·1 % were bars). Twenty-three public PA facilities were evaluated, of which 12 (52·2 %) were squares. In 2015, 677 traffic accidents (57·6 % with victims) and 1312 crimes (81 % thefts/robbery) were recorded in Viçosa (Table 1). All environmental characteristics showed higher densities around schools in the downtown area and in areas with higher neighbourhood income (Fig. 3).

**Table 1:** General characteristics of predominantly ultra-processed food (UPF) stores, public physical activity facilities, traffic accidents and crimes in whole city. Viçosa, Minas Gerais, Brazil, 2015

Characteristics	*n*	%
Predominantly UPF stores		
Bars and beverage distributors	147	55·1
Cafeteria	68	25·5
Ice cream shop	23	8·6
Mobile unhealthy food vendors	13	4·9
Candy stores	10	3·7
Convenience stores	6	2·2
Public physical activity facilities		
Square	12	52·2
Construction site	6	26·2
Multisport court/soccer field/sports square	4	17·3
Park	1	4·3
Traffic accidents with victim		
Yes	390	57·6
No	287	42·4
Crimes		
Theft/robbery	1063	81·0
Bodily injury	209	15·9
Murder	26	2·0
Rape/extortion/kidnapping and false imprisonment	13	1·1

In a multiple linear regression model, the density of predominantly UPF stores was positively associated with total body fat (*β*: 0·12; 95 % CI 0·06, 0·18), while the densities of PA facilities (*β*: –0·08; 95 % CI –0·13, –0·03) and green spaces (*β*: –0·10; 95 % CI –0·16, –0·04) were inversely associated. The densities of predominantly UPF stores (*β*: 0·28; 95 % CI 0·13, 0·43) and green spaces (*β*: –0·18; 95 % CI –0·32, –0·04) had, respectively, a positive and an inverse association with android body fat. The densities of PA facilities (*β*: –0·72; 95 % CI –1·37, –0·08) and crime (*β*: –1·35; 95 % CI –2·02, –0·68) were inversely associated with leptin concentrations. The traffic accidents and green spaces had a positive and an inverse association with concentrations of adiponectin (*β*: 0·35; 95 % CI 0·12, 0·58; *β*: –0·34; 95 % CI –0·52, –0·16, respectively) and RBP4 (*β*: 0·21; 95 % CI 0·10, 0·32; *β*: –0·10; 95 % CI –0·16, –0·03, respectively). No environmental factors were associated with chemerin (Table 2).

**Table 2:** Association of environmental characteristics around urban schools with body fat and adipokine concentrations in children. Viçosa, Minas Gerais, Brazil, 2015

Environmental characteristics	Total fat (%)*		Android fat (%)*		Leptin (ng/ml)†		Adiponectin (µg/ml)†		RBP4 (µg/ml)†		Chemerin (ng/ml)†	
	*β*	95 % CI	*β*	95 % CI	*β*	95 % CI	*β*	95 % CI	*β*	95 % CI	*β*	95 % CI
Predominantly UPF stores (> 50th percentile)	**0·12**	**0·06, 0·18**	**0·28**	**0·13, 0·43**	−0·12	–0·97, 0·72	−0·10	–0·34, 0·14	0·07	–0·03, 0·17	0·29	–0·13, 0·71
Public physical activity facilities (> 50th percentile)	−**0·08**	**–0·13, –0·03**	−0·08	–0·19, 0·04	**−0·72**	**–1·37, –0·08**	0·13	–0·07, 0·33	−0·02	–0·10, 0·05	−0·30	–0·72, 0·12
Green spaces (> 50th percentile)	**−0·10**	**–0·16, –0·04**	**−0·18**	**–0·32, –0·04**	−0·56	–1·19, 0·08	**−0·34**	**–0·52, –0·16**	**−0·10**	**–0·16, –0·03**	−0·27	–0·54, 0·00
Walkability (> 50th percentile)	−0·04	–0·12, 0·05	0·03	–0·15, 0·21	−0·44	–1·37, 0·50	−0·14	–0·37, 0·08	0·02	–0·07, 0·11	0·08	–0·34, 0·51
Traffic accidents (> 50th percentile)	0·04	–0·04, 0·13	0·04	–0·17, 0·25	0·64	–0·01, 1·29	**0·35**	**0·12. 0·58**	**0·21**	**0·10, 0·32**	−0·04	–0·29, 0·20
Crime (> 50th percentile)	0·01	–0·07, 0·09	−0·02	–0·22, 0·17	**−1·35**	**–2·02, –0·68**	−0·17	–0·40, 0·07	−0·07	–0·17, 0·04	−0·20	–0·46, 0·05

UPF, ultra-processed food; RBP4, retinol-binding protein 4.

The category of environmental characteristics with values ≤ 50th percentile was considered as reference.

Linear regression models were performed with generalised estimating equations.

Bold values (*P* < 0·05).

* Adjusted for age, sex, screen time, per capita income, mother´s BMI, school location and neighbourhood per capita income.

† Adjusted for age, sex, screen time, % body fat, per capita income, mother’s BMI, school location and neighbourhood per capita income.

## Discussion

This cross-sectional study has the novelty of showing the relationship between the characteristics of the obesogenic environment and non-traditional biomarkers of obesity in children, such as body adiposity assessed by DXA and pro-inflammatory adipokines (leptin and RBP4). Based on our understanding of the combination of causal factors related to obesity^(3)^, identifying key elements of the environment may help support the elaboration of strategies for the prevention and treatment of obesity in the short and long term.

The built food environment has been highlighted as one of the main determining factors for food choices, since the presence of cafeterias, pizzerias and convenience stores near to schools may favour the purchase of unhealthy foods^(30)^. Our results showed that a higher density of predominantly UPF stores around schools was associated with a higher total and android body fat. Similarly, the presence of ready-to-eat food sales within an 800 m buffer around schools was associated with obesity (assessed by BMI for age) in adolescents from Belo Horizonte, Brazil^(31)^. In addition, a higher density of fast-food outlets and the number of mobile unhealthy food vendors around schools were associated with a higher BMI in children from Canada and Mexico, respectively^(6,18)^. A study that evaluated the availability of food stores around schools in Viçosa, Brazil, showed that all schoolchildren were exposed to sales of unhealthy foods (convenience store; grocery store; bar; chocolate store; beverage distributors; snack bar; ice cream shop and mobile unhealthy food vendors), regardless of neighbourhood income and school location^(32)^. In Brazil, although bars mostly sell alcoholic beverages, they are accessible to children as they also sell soft drinks, snacks and sandwiches, which are consumed by these people. This fact justifies the inclusion of bars in Brazilian investigations about predominantly UPF stores^(33,34)^.

Little is known about the relationship between the food environment and obesity-related inflammatory markers. However, a previous research found that a pro-inflammatory diet, characterised by high consumption of UPF, was associated with higher chemerin concentration (pro-inflammatory adipokine) and lower concentration of adiponectin (anti-inflammatory adipokine)^(35)^. Children (mean age: 7·9 years) from Massachusetts, USA, living in areas with higher access to healthy food, showed lower C-reactive protein during adolescence (mean age: 13·1 years)^(7)^. Consumption of UPF, which can be rich in sugars and saturated fats, may trigger an innate immune response, with increased secretion of pro-inflammatory compounds, such as adipokines and C-reactive protein^(36)^.

Moreover, we found an inverse association of public PA facilities with total body fat and leptin concentration. In adolescents from Taiwan, PA facilities around schools were associated with lower abdominal adiposity measurements^(15)^. In contrast, in Canada, there was no association between recreation opportunities around schools and BMI z-score in adolescents aged 10–14 years^(6)^. The lack of studies that evaluated the inflammatory markers limits the understanding of this relationship in children. However, it is known that lower levels of PA or sedentary time in childhood are associated with increased leptin, which is a pro-inflammatory adipokine^(37,38)^. Therefore, further studies are needed to investigate the relationship of environmental characteristics with pro-inflammatory adipokines.

Similarly, our results showed that green spaces were inversely associated with total and android body fat, and RBP4 concentration. In contrast, a study with children from Porto, Portugal, demonstrated an association between the built environment (construction sites, land without current use and railways), BMI and body fat around schools, but no association was found for green areas^(13)^. The higher density of green spaces around schools has been related to less exposure to traffic-related air pollutants^(39)^. These pollutants have been associated with increased adiposity and inflammation risk by various mechanisms, such as modifying adipocyte differentiation, eating behaviour regulation, metabolic homoeostasis and disorders in the gastrointestinal tract and microbiota composition^(40)^. Moreover, green spaces may reduce obesity and inflammation by motivating the practice of recreational and/or physical activities once they indicate the presence of open areas favourable to outdoor and healthy habits and, thus, reducing sedentary behaviour^(41)^.

Traffic accidents were positively associated with RBP4 concentration. Contrary to our hypothesis, traffic accidents were positively associated with adiponectin, and crime was inversely associated with leptin concentration. There is a lack of investigations between these environmental characteristics and adipokines in the pediatric population, and more studies are necessary. However, evidence has shown the higher densities of traffic accidents and crime are characteristics of an unsafe environment, and it can reduce the active transport form and the access and/or availability to food stores, and health-promoting spaces^(42,43)^. Violent crime around schools was directly associated with obesity in children from California, USA^(44)^. In contrast, a study conducted in Spokane, USA, showed no association between traffic accidents around schools and obesity in children^(45)^. In recent years, Viçosa has seen an increase in crime, with a murder rate higher than the state average^(46)^. Compared to other cities of the same size, Viçosa has a higher ratio of vehicles/population, in addition to the poor quality of the roads, such as problems with sizing, paving and signalling^(47)^. Thereby, urban traffic is related to perceived safety, affecting the motivation to practice outdoor activities and active transport mode^(8,42)^.

In the final model, there was no association between walkability and body fat and adipokine concentrations. The relationship between walkability and health outcomes appears to be still controversial^(48)^. In a Canadian study, walkable school neighbourhoods were not associated with active travel to school and were inversely related to free play in children^(49)^. Although the walkability index used considers important characteristics that favour walking (residential and commercial densities, intersections between roads, the presence of sidewalks and public lighting), other individual and environmental factors may be considered, such as slope, parents’ perception of neighbourhood safety, aesthetics of the landscape and the quality of stores along the street^(48,50)^. As well as walkability, characteristics such as traffic accidents and crimes had a higher density downtown, which can lead to a perception of insecurity by parents and, consequently, modify the associations between walkability and children’s health outcomes. In addition, more than half of the children in the sample used a transport vehicle to school (data not shown). Thus, we highlight the importance of walkability as a motivator for children and their guardians to adopt walking as a transport mode to reduce traffic-related air pollution, noise and sedentary behaviour.

Our work has several strengths, such as: (1) this is one of the few studies on urban environment carried out in a middle-income country, which evaluated the association of combined environmental characteristics; (2) according to our knowledge, this is the first epidemiological study to assess the relationship of the urban environment around schools with adiposity (measured by DXA, a reference method) and adipokine concentrations in children; (3) in the studied city, economic, cultural and social activities are concentrated in downtown and, for this reason, we adjusted the analysis for the school location. Environmental characteristics, such as green spaces, walkability and neighbourhood income, were obtained from the 2010 Census database^(26)^. For this reason, there is a time difference compared with data collection (2015–2016). However, no changes were observed in the city that could impact a difference in physical structures in this period (data not shown). In addition, data obtained from 2010 Census are high-quality information, allowing the comparison between different Brazilian cities and replicating analyses in future works. As limitations, this is a cross-sectional study, which did not allow us to evaluate the causality among environmental characteristics, body fat and adipokines. Additionally, there is a lack of consensus on the cut-off point for android fat and adipokines to predict abdominal obesity and low-grade inflammation among children, respectively.

In conclusion, characteristics of the obesogenic environment around schools were associated with total and android body fat and adipokine concentrations in Brazilian children from a medium-sized city. Public policies are necessary to create and/or improve the availability and/or access to a safe environment that promotes healthy choices of eating habits and lifestyle and, consequently, be one of the strategies to reduce body fat and low-grade inflammation associated in children.

## Supplementary material

For supplementary material accompanying this paper visit https://doi.org/10.1017/S1368980023001696. click here to view supplementary material
