# Supplementary material for: Characteristics of the obesogenic environment around schools are associated with body fat and low-grade inflammation in Brazilian children
Source: Public Health Nutr. 2023 Aug 10;26(11):2407–17. doi: 10.1017/S1368980023001696 (PMC10641627; doi:10.1017/S1368980023001696)
Supplement: Supplementary file 1 [file S1368980023001696sup001.pdf]

Supplementary Material 1. Grouping of types of food stores, according to the degree of processing of most commercialized foods.

**Group**	**Food store**	**Characteristics**
Predominantly ultra-processed food stores	Bars	Food services that offer a wide range of alcoholic beverages, in addition to offering quick meals and sandwiches, with full table service or ordering at the counter. In Brazil, bars are accessible to children as they also sell soft drinks, snacks, and sandwiches, which are consumed by these people.
	Beverage distributors	Companies that sell beverages (alcoholic or not) to consumers or other food stores, without a consumption area.
	Cafeteria	Food services that offer quick meals, mainly sweets, snacks, sandwiches, and a wide variety of beverages (juices, coffees, teas, soft drinks, and alcoholic beverages in smaller quantities), with full table service or orders at the counter.
	Ice cream shop	Food services that offer ice cream, smoothies, *açaí*, frozen yogurt, as main items, with consumption area on store.
	Mobile unhealthy food vendors	Food services that offer prepared food, such as sweets and snacks, for immediate consumption, in open places, permanent or not.
	Candy stores	Food services that offer chocolate, candy, gum, lollipops, and other sweets as main items, with no consumption area on store.
	Convenience stores	Stores that offer predominantly industrialized food products in addition to other non-food products, with no consumption area on store.

Source: Adapted from Duran (2013); CAISAN (2018); SEBRAE (n.d.).
